# Exploring Long-Term Anomalies in the Vegetation Cover of Peri-Urban Parks Using the Fisher-Shannon Method

**DOI:** 10.3390/e24121784

**Published:** 2022-12-06

**Authors:** Luciano Telesca, Angelo Aromando, Farid Faridani, Michele Lovallo, Gianfranco Cardettini, Nicodemo Abate, Giancarlo Papitto, Rosa Lasaponara

**Affiliations:** 1 Institute of Methodologies for Environmental Analysis, National Research Council, 85050 Tito, Italy; 2 DICEM, Department of European and Mediterranean Cultures, Environment, and Cultural Heritage, University of Basilicata, 85100 Potenza, Italy; 3 Agenzia Regionale per la Protezione dell’Ambiente della Basilicata, 85100 Potenza, Italy; 4 Institute of Heritage Science (CNR—ISPC), National Research Council, C.da S. Loja, 85050 Tito, Italy; 5 Arma dei Carabinieri, Comando Unità Forestali, Ambientali e Agroalimentari, Via. G. Carducci 5, 00187 Roma, Italy

**Keywords:** Fisher-Shannon method, satellite time series, vegetation

## Abstract

The main goal of this study was to evaluate the potential of the Fisher-Shannon statistical method applied to the MODIS satellite time series to search for and explore any small multiyear trends and changes (herein also denoted as inner anomalies) in vegetation cover. For the purpose of our investigation, we focused on the vegetation cover of three peri-urban parks close to Rome and Naples (Italy). For each of these three areas, we analyzed the 2000–2020 time variation of four MODIS-based vegetation indices: evapotranspiration (ET), normalized difference vegetation index (NDVI), leaf area index (LAI), and enhanced vegetation index (EVI). These data sets are available in the Google Earth Engine (GEE) and were selected because they are related to the interactions between soil, water, atmosphere, and plants. To account for the great variability exhibited by the seasonal variations while identifying small multiyear trends and changes, we devised a procedure composed of two steps: (i) application of the Singular Spectrum Analysis (SSA) to each satellite-based time series to detect and remove the annual cycle including the seasonality and then (ii) analysis of the detrended signals using the Fisher-Shannon method, which combines the Shannon entropy and the Fisher Information Measure (FIM). Our results indicate that among all the three pilot test areas, Castel Volturno is characterized by the highest Shannon entropy and the lowest FIM that indicate a low level of order and organization of vegetation time series. This behaviour can be linked to the degradation phenomena induced by the parasite (*Toumeyella parvicornis)* that has affected dramatically the area in recent years. Our results were nicely confirmed by the comparison with in situ analyzed and independent data sets revealing the existence of subtle, small multiyear trends and changes in MODIS-based vegetation indices.

## 1. Introduction

Natural capital (NC) through the ecosystem process provides ecosystem services that are vital and critical to the functioning of the Earth’s life-support system, such as (but not only) air, water, fertile soil, pollination, and hazard protection. The concept of NC and associated ecosystem services reflects a recognition that environmental systems are fundamental not only for providing resources and services but also contributing to economic outputs and social well-being. The biophysical evaluation of the ecosystem then leads directly to the ecological and monetary evaluation, since it depends on its state of conservation. Nevertheless, if any form of vegetation cover (cropland, grassland, forest, etc.) provides numerous ecosystem services the estimation of the status and trends of natural capital poses critical challenges due to the diversity of environmental assets, stocks, and flows. Moreover, it is widely recognized that today climate change and anthropogenic pressures do alter major geophysical conditions and adversely affect NC and ecosystem services, accelerating their depletion. NC assets are limited and vulnerable, and irreversible environmental changes may render impossible the replacing of NC assets that must be preserved, and this requires its constant assessment and systematic monitoring.

A recent report by the European Environmental Agency (EEA) underlined the importance of Earth Observation (EO) data for the monitoring and accounting of the natural capital to support political decisions, especially for the most critical environmental conditions.

The use of EO-based indicators is particularly relevant for environmental monitoring because RS (remote sensing)-derived data have been shown to be useful across many fields, at different temporal and spatial scales from global to local levels using open data and tools from NASA (MODIS, TM) and ESA (Sentinel 1–5), acquired systematically and available for the whole globe. Moreover, the rapidly increasing developments of the EO and Information and Communication Technologies (ICT), including cloud-based resources, strongly facilitate and support the massive increase of the use of satellite data for change detection and vegetation monitoring, including risk analyses. Recently, the availability of big and open data from a cloud source tool, such as Google Earth Engine, strongly facilitates the use of satellite data such as those available from NASA (MODIS, Landsat satellites etc.) and ESA (Sentinels) for risk monitoring and hazard mitigation. Cloud-based computing systems provide ready-to-use and up-to-date datasets along with impressive computing power without the need to download and locally store large amounts of data.

The EO-based indicators, as the well-known vegetation indices, range from spectral indices such as the Normalized Difference Vegetation Index (NDVI), to biophysical variable estimates such as the Leaf Area Index (LAI), fraction of absorbed photosynthetically active radiation (absorbed by the photosynthesizing tissue in a canopy) (FAPAR), and fraction of green vegetation cover (FCover) [1].

Many studies [2,3,4,5] suggest that evapotranspiration (ET), in combination with other vegetation indices, is an important variable to monitor and estimate crop yield and biomass. ET is the process of transferring water vapor from the Earth’s surface to the atmosphere through evaporation and plant transpiration from wet surfaces. ET plays an important role in the earth-atmosphere interactions, since it connects the energy, water, and carbon cycles [4]. The potential and reference ET are influenced through prevailing weather conditions such as radiation, temperature, wind, and relative humidity [3]. The status of actual evapotranspiration (ETa), in comparison with its long historical records (e.g., the ETa anomaly for a given period), has the potential to identify vegetation stress in time and space [6]; therefore, ETa is an essential element in the design, development, and monitoring of agricultural and environmental systems [4].

By advancing the remote sensing technologies, ET has been consistently estimated at multiple spatiotemporal scales using models that can be grouped into: (I) vegetation index (VI)-based models which rely on vegetation indices (e.g., leaf area index (LAI) or the Normalized Difference Vegetation Index (NDVI)) as well as meteorological inputs (mainly net radiation (Rn), air temperature (Tair), and vapor pressure deficit (VPD)) following the Penman-Monteith logic; and (II) land surface temperature (LST)-based models which rely on LST as an effective proxy for soil moisture following the surface energy balance (SEB) [7]. Some of the well-known VI-based models are the Priestley-Taylor Jet Propulsion Laboratory (PT-JPL) [8], the Moderate Resolution Imaging Spectroradiometer (MODIS) Land Surface Evapotranspiration (MOD16) [9], and the Global Land-Surface Evaporation Amsterdam Methodology (GLEAM) [10]. LST-based models are the Surface Energy Balance Algorithm for Land model (SEBAL) [11], the Mapping Evapotranspiration at High Resolution with Internalized Calibration (METRIC) [12], and the Surface Energy Balance System (SEBS) [13].

Another important means of quantifying drought in a spatially comparable way across different regions is the Palmer Drought Severity Index (PDSI), originally developed by Palmer [14]. Various studies show that PDSI is very effective in determining long-term drought, considering the basic effects of global warming through potential evapotranspiration, and taking precedent (prior month) conditions into account [15,16,17,18]. For the calculation of the PDSI, four inputs are needed: precipitation, temperature, latitude, and the soil’s available water capacity (AWC) of the study area, which is a constant also known as the field capacity [19]. These four inputs are used to compute a water balance for the study area, which then serves as the basis for the calculation of the PDSI. For a detailed explanation of the calculation of the PDSI, refer to [20].

The main goal of this study was to evaluate the potential of the Fisher-Shannon statistical method to explore any anomalies happening for the vegetation cover around large urban areas using soil-water-atmosphere-plant-related satellite products available in the Google Earth Engine cloud database (i.e., LAI, NDVI, EVI, and ET from MODIS). Periurban parks were selected for our investigations as particularly significant areas because they play a key role not only in improving environmental quality and life but also in facing climatic change and mitigating climate change effects.

## 2. Study Areas and Dataset

**Figure 1:**
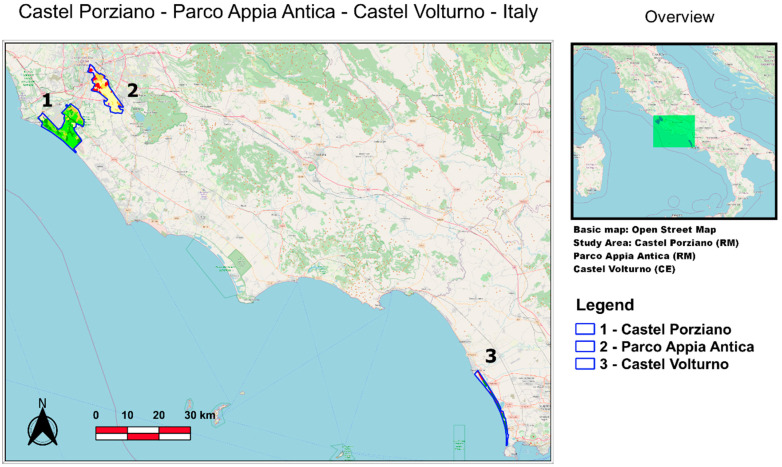
Locations of the investigated areas: Castel Porziano (1, green shaded area); Parco Appia Antica (2, yellow shaded area); and Castel Volturno (3).

For the purpose of this study, the following three study areas in Italy were selected: Appia Park and Castel Porziano in the center, and Castel Volturno in the south (Figure 1). These areas were selected because they are representative of diverse vegetation covers, as detailed in the following Section 2.1, Section 2.2, Section 2.3.

The characteristics of the study areas (including longitude, latitude, area, annual precipitation, annual mean temperature, vegetation description, and climate system) are presented in Table 1.

### 2.1. Castel Volturno

**Figure 2:**
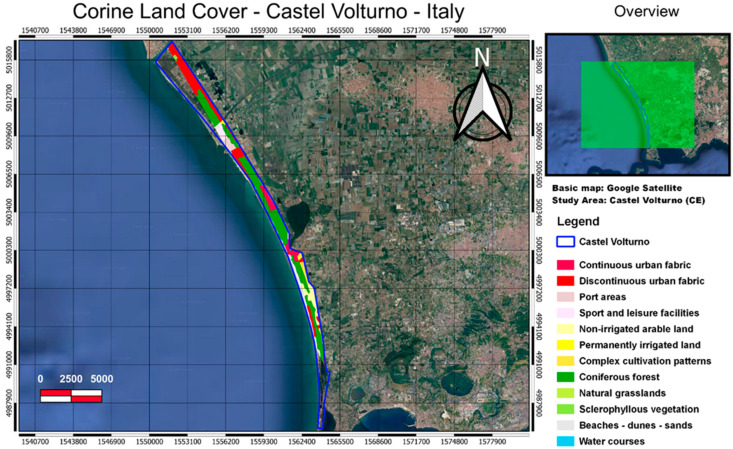
The Castel Volturno site occupies an area of around 268 hectares, mainly characterized by the presence of woods holm oak, pine forests with Pinus pinea, and a nucleus of retrodunal hygrophilous vegetation. The land use classes are from the Corine land cover.

Castel Volturno is a natural reserve which occupies a total area of 268 hectares; it extends along the sandy coast of the municipality of Castel Volturno (CE), in a strip between the mouth of the Regi Lagni to the north and the mouth of Lago Patria to the South (Figure 2). The site includes the protected area named ZSC IT8010021 “Pineta di Patria” and the Regional Nature Reserve “Foce Volturno-Costa di Licola” made up of pines.

### 2.2. Castel Porziano

**Figure 3:**
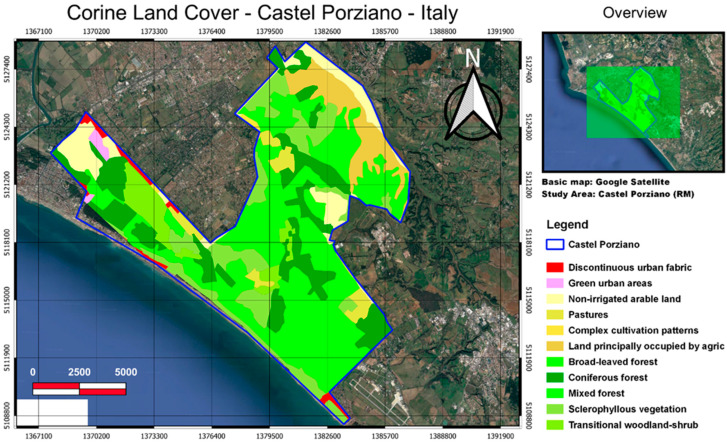
The Castel Porziano site occupies an area of around 268 hectares, mainly characterized by the presence of holm oak (261 hectares), cork oak wood (460 hectares), and stone pine forest (750 hectares). The woods alternate between clearings and natural grasslands. The land use classes are from the Corine land cover.

The Presidential Estate of Castelporziano is about 25 km from the center of Rome (Figure 3) and covers an area of 60 km^2^ (6039 hectares), consisting of humid areas behind the dunes and areas with low and high scrub with the typical evergreen and aromatic species. Most of the extension is occupied by lowland hygrophilous wood (lowland wood linked to humid environments), characterized by the presence of evergreen and deciduous oaks and by more purely hygrophilous species, near the wetlands. The peculiarity of Castelporziano is above all linked to the interpenetration of the oak grove typical of the Mediterranean climate and the oak grove typical of the continental climate. Among the evergreen oaks, the holm oak, the cork oak, and the crenata oak, a hybrid between turkey oak and cork oak, are widely diffused. Among the deciduous oaks we note the turkey oak, the English oak, and the farnetto, while in the cooler wetlands we can find poplar, ossifillo ash, maple, hornbeam, and Oriental hornbeam typical of Mediterranean coastal environments. The wood (mixed plain), one of the most delicate ecosystems to be protected, extends for about 2300 hectares; the Mediterranean scrub environments, low and high, cover an area of about 500 hectares; the holm oak occupies an area of 261 hectares above all in the back dune area; and the cork oak wood covers an area of about 460 hectares. The woods alternate between clearings and natural grasslands, forming plant associations of great environmental variety. The stone pine forests, created with artificial reforestation, extend for about 750 hectares with the purpose of consolidating the sandy dunes and protecting the rear dunes from sea winds.

### 2.3. The Appia Antica Regional Park

**Figure 4:**
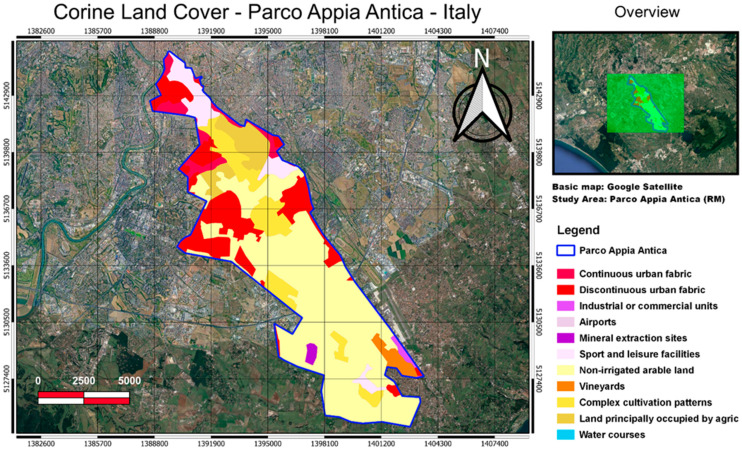
The Appia Park is around 4580 hectares in size. It is a mosaic of various environments: large spaces intended for cultivation and extensive grazing are interrupted by uncultivated areas; residual wooded strips, where agricultural exploitation has not arrived or has long since ceased; ditches with the presence of riparian vegetation; and some wet areas. The land use classes are from the Corine land cover.

The Appia Antica Regional Park, with its 4580 hectares, is the largest urban protected area in Europe. A green wedge runs from the city center towards the Castelli Romani (Figure 4). This green wedge, a vast 4580 hectares (following the last extension in October 2018), is characterized by various areas of interest: the Via Appia Antica and its adjacencies; the Caffarella Valley; the archaeological area of the Via Latina and of the Aqueducts; the Tenuta di Tormarancia; the Tenuta Farnesiana; and the areas of Divino Amore, Falcognana and Mugilla. The park is so vast that it affects three municipalities: those of Rome, Ciampino, and Marino. The Park today looks like a mosaic of different environments: large spaces intended for cultivation and extensive grazing are interrupted by uncultivated areas; residual wooded strips, where agricultural exploitation has not arrived or has long since ceased; ditches with the presence of riparian vegetation; and some wet areas. These seminatural environments and the agricultural contexts now represent the agroecosystem of the Roman countryside. It is a system of considerable naturalistic and scientific interest, due to the presence of wildlife communities and plant associations, consistent with the ecological potential of the area. The Appia Antica Park is a substantial part of the Ecological Network of the city of Rome and is the most important protected periurban area of the Lazio Region.

### 2.4. Data Sets

Four different satellite products available in the cloud storage of Google Earth Engine were selected because they are related to the interactions between soil, water, atmosphere, and plants. For the purpose of this study, MODIS products were chosen due to their global coverage and long duration of data acquisition. The Supplementary File summarizes the characteristics of the studied datasets. The area-averaged time-series of studied parameters were extracted for the polygons representing the study regions (see Figure 1, Figure 2, Figure 3 and Figure 4) using the GEE JavaScript API for the common period of 2001–2020.

## 3. Methods

### 3.1. The Singular Spectrum Analysis

There are several techniques for decomposing a time series into a certain number of independent components; among these, Singular Spectrum Analysis (SSA) [21] represents an efficient and well-known decompositional method that is based on phase-lagged copies of the series. The independent components obtained by applying the SSA can be easily recognizable as slowly changing trend, oscillatory components, and structureless noise [22].

Let us consider a time series *y_i_* (*i* = 1, …, *N*) and a lag *M*; then the Toeplitz lagged correlation matrix can be constructed:

(1) $$c_{ij}=\frac{1}{N-\left|i-j\right|}\sum_{k=1}^{N-\left|i-j\right|}y_{k}y_{k+\left|i-j\right|},\;1\leq i,j\leq M$$

Sorting its eigenvalues *λ_k_* in decreasing order, the corresponding eigenvectors *E_k,j_* where *j* and *k* vary from 1 to *M* are used to calculate the *k*-th principal component *i*

(2) $$a_{ik}=\sum_{j=1}^{M}y_{i+j}E_{jk},\;0\;\leq \;i\leq \;N-M,$$

and the *k*-th reconstructed component of the time series:

(3) $$R_{k}=\frac{1}{M}\sum_{j=1}^{M}a_{i-j,k}E_{jk},\;M\leq \;i\leq \;N–M+1$$

Since the eigenvalue *λ_k_* represents the fraction of the total variance of the original series explained in the *k*-th reconstructed component *R_k_*, the decreasing order of the eigenvalues also reflects the decreasing order of the reconstructed components by the fraction of the total variance of the series [23]. SSA requires that the lag *M* is properly selected. Khan and Poskitt [24] calculated the maximum *M* = (log *N*)*^c^*, 1.5 ≤ *c* ≤ 2.5.

The minimum description length (MDL) criterion [25]:

(4) $$MDL(k)\;=\;-\log{\left(\frac{\overset{p}{\underset{i=k+1}{\Pi }}\lambda_{i}^{\frac{1}{p-k}}}{\frac{1}{p-k}\sum_{i=k+1}^{p}\lambda_{i}}\right)}^{(p-k)N}+\frac{1}{2}k(2p-k)\log N$$

is used to separate the series into two parts that we can define as trend and detrended series; *λ_k_* are the eigenvalues, *p* is the number of eigenvalues, identical to *M*, and *N* is the length of the original series. The separation occurs at the value of *k* ∈ {0, 1, 2, …, *p* − 1} for which the MDL is minimized.

### 3.2. The Fisher-Shannon Method

By the Fisher-Shannon method, the informational properties of a time series can be investigated, namely the Fisher Information Measure (FIM) and Shannon entropy (SE), which are used to quantify the local and global smoothness of the distribution of a series. The FIM and SE can be employed to characterize the complexity of non-stationary time series described in terms of order and organization [26]. The FIM measures the order and organization of the series, and the SE its uncertainty or disorder [27]. The FIM and SE are defined by the following formulae:

(5) $$\mathrm{FIM}=\int_{-\infty }^{+\infty }{\left(\frac{\partial }{\partial x}f(x)\right)}^{2}\frac{dx}{f(x)},$$

(6) $$\mathrm{SE}=-\int_{-\infty }^{+\infty }f_{X}(x)\log f_{X}\left(x\right)dx$$

where *f*(*x*) is the distribution of the series *x*. The Shannon entropy power *N_X_* is generally used instead of SE:

(7) $$N_{X}=\frac{1}{2\pi e}e^{2H_{X}}$$

to avoid dealing with negative quantities. FIM and *N_X_* are not independent of each other due to the isoperimetric inequality FIM⋅*N_X_* ≥ *D* [28], where *D* is the dimension of the space, which, for the time series, is 1.

FIM and *N_X_* depend on *f*(*x*), whose accurate estimation is crucial in order to obtain reliable values of informational quantities. For calculating FIM and *N_X_,* we applied the kernel-based approach that Telesca and Lovallo [29] demonstrated to be better than the discrete-based approach. Thus, we apply the kernel density estimator method for *f(x)* [30,31] as shown in the following formula:

(8) $${\hat{f}}_{M}(x)=\frac{1}{Mb}\sum_{i=1}^{M}K\left(\frac{x-x_{i}}{b}\right)$$

where *M* and *b* denote the length of the series and the bandwidth, respectively, while *K(u)* is the kernel that is a continuous, symmetric, and non-negative function satisfying the two following constraints:

(9) $$K\left(u\right)\geq 0\;\mathrm{and}\;\int_{-\infty }^{+\infty }K(u)du=1$$

*f*(*x*) is estimated by means of an optimized integrated procedure using the algorithms of Troudi et al. [32] and Raykar and Duraiswami [33], with a Gaussian kernel:

(10) $${\hat{f}}_{M}(x)=\frac{1}{M\sqrt{2\pi b^{2}}}\sum_{i=1}^{M}e^{-\frac{{\left(x-x_{i}\right)}^{2}}{2b^{2}}}$$

Due to the isoperimetric inequality, the Fisher-Shannon information plane (FSIP), which has the *N_X_* as the x-axis and FIM as the y-axis, represents a very useful tool to investigate the time dynamics of signals [34]. For scalar signals, the curve FIM∙*N_X_* = 1 separates the FSIP into two parts, and each signal can be represented by a point located only in the space FIM*∙N_X_* > 1.

## 4. Results

We analyzed the 2000–2020 time variation of four vegetation indices: evapotranspiration (ET), normalized difference vegetation index (NDVI), leaf area index (LAI), and enhanced vegetation index (EVI).

First, the SSA was applied to each time series, and the value of the phase lag *M* was selected, taking into account the sampling time of the series (8 days for ET and LAI; 16 days for EVI and NDVI). To detect at least the annual cycle, *M* was set as 45 for the ET and LAI series, and as 24 for the EVI and NDVI series; moreover, these values fit well with Khan and Poskitt’s [24] criterion, varying the length of the data from 503 values (EVI and NDVI) to 965 (ET) and 1003 (LAI).

**Figure 5:**
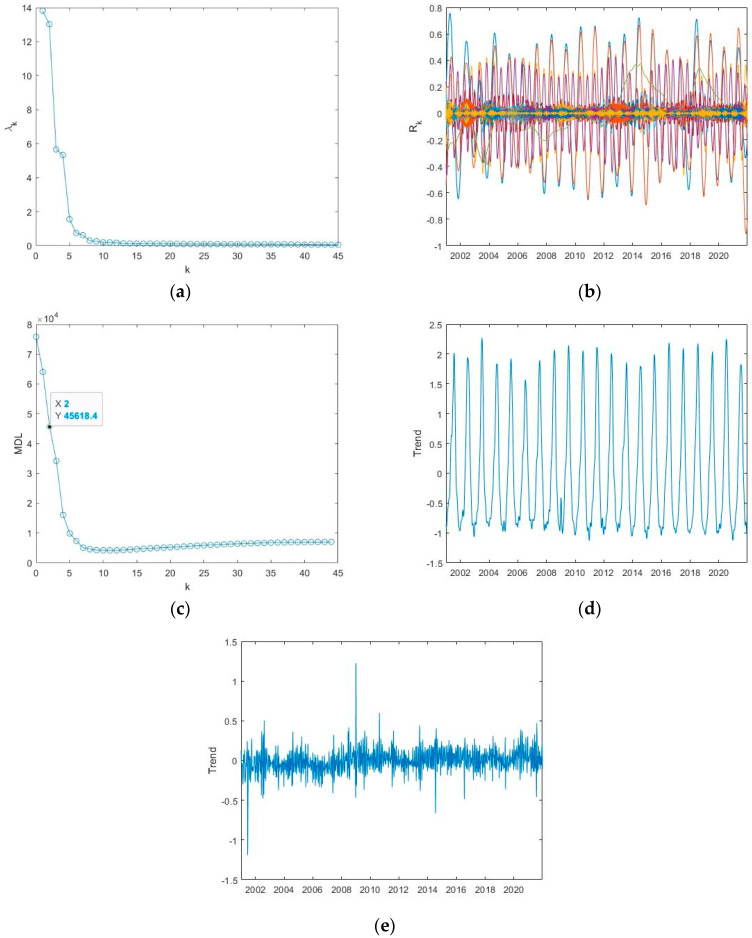
Application of the SSA to the ET series of Appia: (**a**) eigenvalue spectrum; (**b**) reconstructed components; (**c**) MDL versus the number of components *k*; the minimum MDL is at *k_min_* = 11; (**d**) trend; (**e**) detrended series.

Figure 5 shows the application of the SSA to the ET time series of Appia as an example. Before applying the SSA, the original time series was normalized. Figure 5a shows the eigenvalue spectrum of the SSA decomposition; each eigenvalue corresponds to a reconstructed component and represents the fraction of the total variance of the original series explained by that component. Figure 5b shows all the obtained reconstructed components, whose behaviour varies from oscillatory with amplitude modulation to apparently noisy.

**Figure 6:**
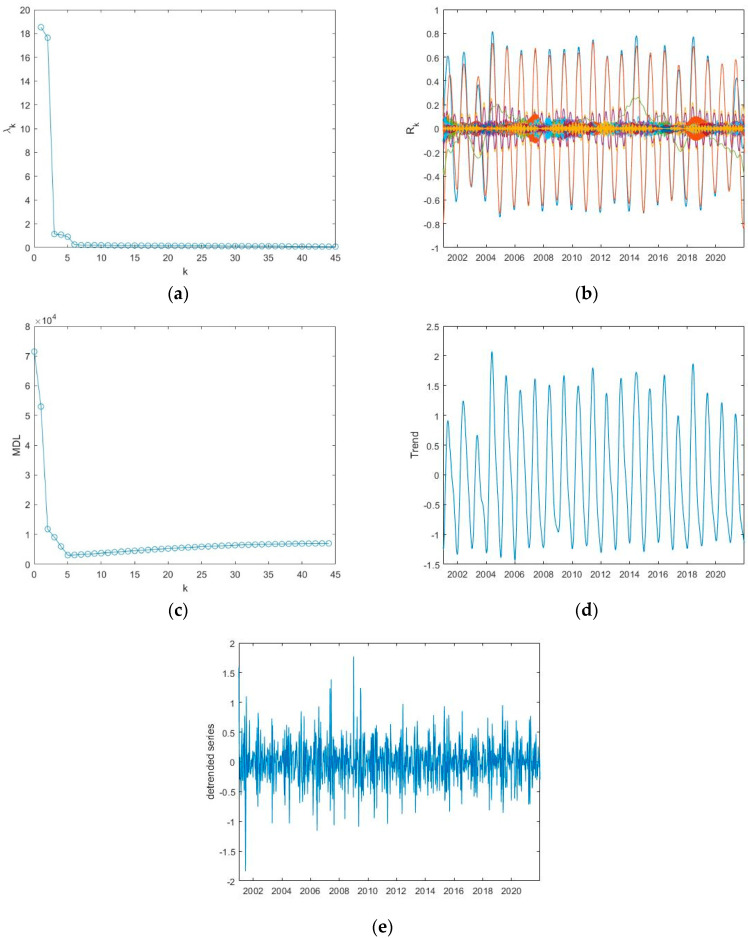
Application of the SSA to the ET series of Volturno: (**a**) eigenvalue spectrum; (**b**) reconstructed components; (**c**) MDL versus the number of components *k*; the minimum MDL is at *k_min_* = 5; (**d**) trend; (**e**) detrended series.

**Figure 7:**
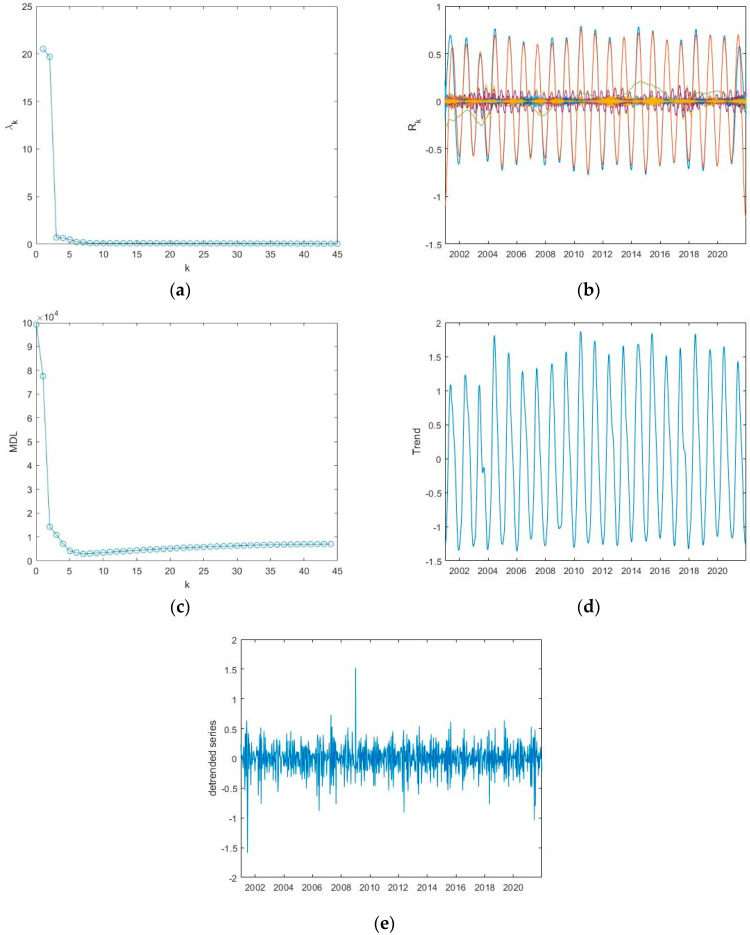
Application of the SSA to the ET series of Porziano: (**a**) eigenvalue spectrum; (**b**) reconstructed components; (**c**) MDL versus the number of components *k*; the minimum MDL is at *k_min_* = 7; (**d**) trend; (**e**) detrended series.

Applying the MDL criterion, the signal is separated into a trend and a detrended series; the value of *k_min_* corresponding to the minimum MDL represents the number of the first reconstructed components to sum up for obtaining the trend (Table 2). Applying this criterium to the ET time series of Appia, the MDL curve is shown in Figure 5c, and the minimum MDL is at *k_min_* = 11; thus, the trend is obtained by summing up the first 11 reconstructed components (Figure 5d) and the detrended series by subtracting the trend from the original normalized series (Figure 5e). Figure 6 and Figure 7 show, similarly to Figure 5, the application of SSA to the ET series of the other two sites, Castel Volturno and Castel Porziano.

The trend is characterized by an oscillatory behaviour that explains the seasonal cycles of the series, very likely linked with the meteoclimatic variability. The detrended series, although apparently noisy, would represent the inner time dynamics of the series that might be not influenced by external driving mechanisms. Table 2 shows for all the investigated indices the value of the minimum of the MDL criterion.

Our aim is to characterize the time dynamics of the inner vegetation of the investigated sites by using the Fisher-Shannon method. Thus, for each site we focused on the detrended series, since this represents the inner time variability of vegetation not influenced by external meteoclimatic factors.

**Figure 8:**
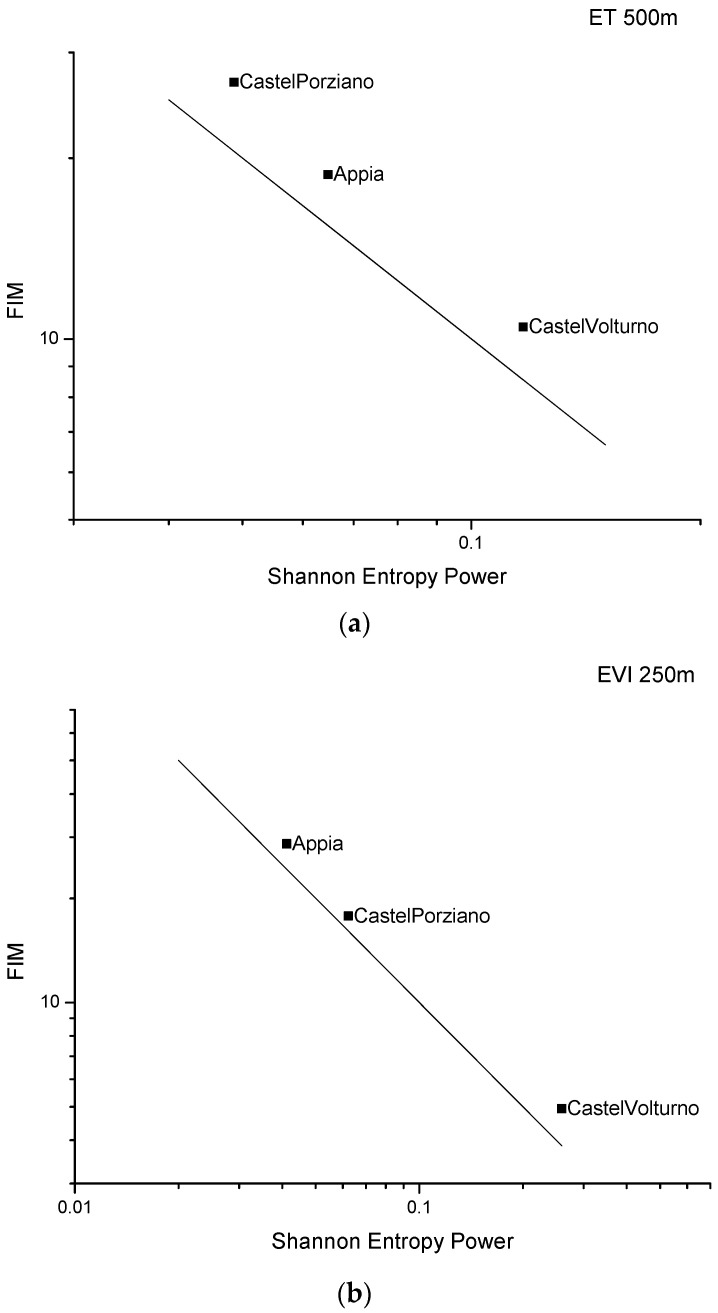

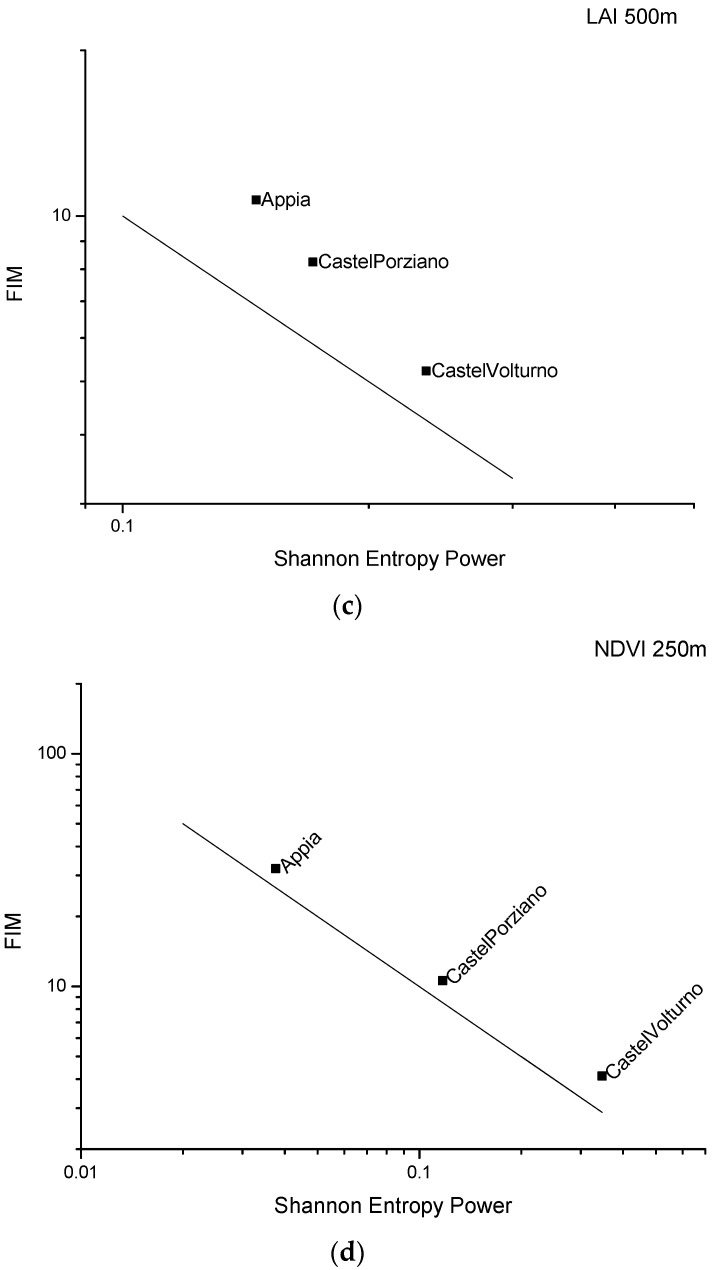
FSIP of ET (**a**), EVI (**b**), LAI (**c**), and NDVI (**d**). The black line in each panel represents the isoperimetric line.

Figure 8 shows the FSIP of ET (Figure 8a), EVI (Figure 8b), LAI (Figure 8c), and NDVI (Figure 8d). The FSIP indicates that the Castel Volturno site is characterized by the highest Shannon entropy power and the lowest FIM that suggests a low level of order and organization of vegetation indices; Appia Park, except for the ET, is characterized by the lowest Shannon entropy power and the lowest FIM that reveal a relative high level of order and organization of vegetation indices; Castel Porziano is generally characterized by an “intermediate” behaviour, since the vegetation indices, except ET, are located in the FSIP between Castel Volturno and Castel Porziano.

## 5. Discussion

The potential of satellite systems for the monitoring of vegetation resources is widely recognized and nowadays the most recent ICT technological developments, the joint use of artificial intelligence and EO, and the growing availability of information (as well as data from the free cloud such as GEE) have opened new frontiers and application fields.

The use of EO-based indicators for the monitoring of vegetation is particularly relevant, and recently the RS-derived data have been shown to be useful across many fields; however, undoubtedly, large earth observation data such as satellite time series pose several challenges to face in order to transform data in useful and reliable information.

For the purpose of our investigation, the four vegetation indices (ET, NDVI, LAI, and EVI) are various satellite products available in the cloud storage of Google Earth Engine, selected because they are related to the interactions between soil, water, atmosphere, and plants, while MODIS products were chosen due to their global coverage and long duration of data acquisition.

The analyses of satellite time series are generally quite complex and time-consuming due to the amount of data, but they are expected to be as suitable for the identification of both slow and fast changes as, for example, parasites or salinization, deforestation or wildfires, which adversely have been affecting NC during the last decades. In reality, the ability and effectiveness of change detection approaches and methods depend on the ability to account for the great variability exhibited by the seasonal variations (at seasonal and/or intra-annual scales) while identifying small multi-year trends and changes at diverse interannual time scales.

The methodological approach consisted of the following steps: decomposition of each satellite vegetation index through the SSA, detection of annual and seasonal cycles, separation between trend and detrended series, and application of the Fisher-Shannon method to the detrended series. In particular, this approach enabled us to perform the deseasonality and, therefore, to split the stronger seasonal dynamics from the subtle inner time variability of the investigated signals. Identifying and extracting information related to the potential presence of small but significant trends or variations in vegetation is an important issue, and the effectiveness of change detection approaches depends on their ability to account for both the great variability exhibited by the seasonal variations and the small multiyear changes that might be completely veiled by the seasonal dynamics.

Our study highlighted that the trend is characterized by an oscillatory behaviour that explains the seasonal cycles of the series, very likely linked with the meteoclimatic variability. Furthermore, the detrended series, whose variability our study has focused on, although apparently noisy, would represent the inner time dynamics of the series that might be not influenced by external driving mechanisms.

For Castel Volturno, a low level of order and organization of the MODIS time series was observed during the whole investigated period. This behaviour denoted an anomalous vegetational dynamic that can be explained and attributable to the effect of attack by the parasite *Toumeyella parvicornis*, which in the recent years adversely impacted the Pinus trees of the area, dramatically damaging them.

**Figure 9:**
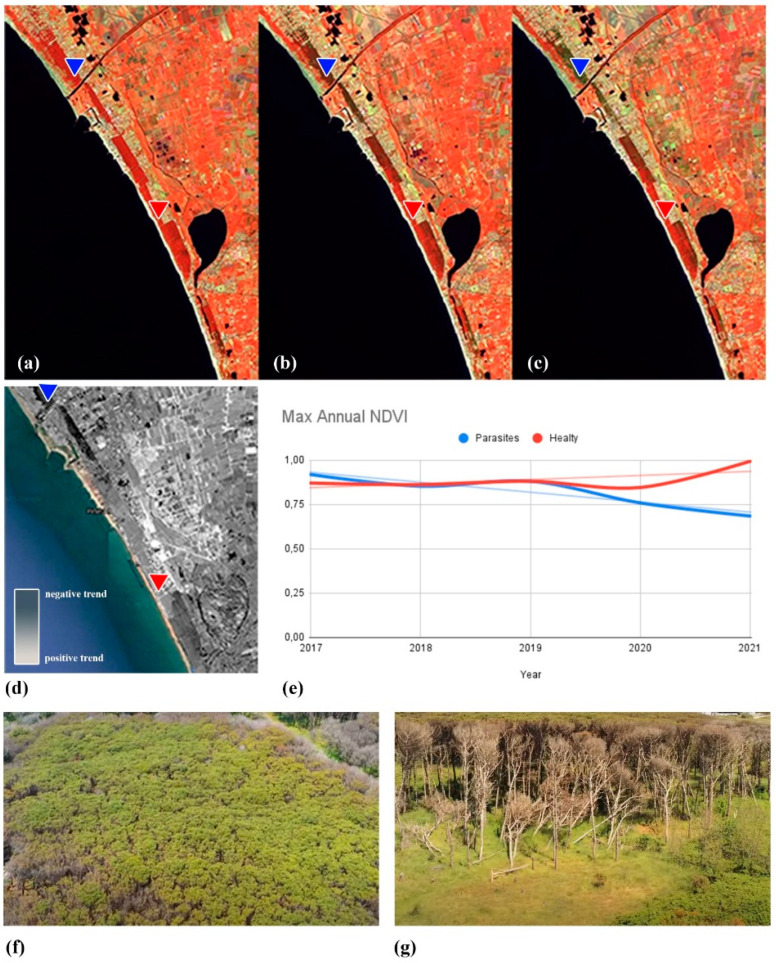
Outputs from the analysis jointly conducted by Consiglio Nazionale delle Ricerche (CNR) and Carabinieri [35] based on the Sentinel 2 NDVI time series. Details related to the results were obtained by the independent analyses on Sentinel 2 ((**a**)—2016, (**b**)—2019, (**c**)—2021) conducted by CNR and verified by Carabinieri by field surveys. The figure shows in a, b, and c false colours RGB (NIR, SWIR, RED) with the healthy vegetation coloured red/orange (red triangle) and the unhealthy vegetation tending towards green (blue triangle). The dark grey areas in (**d**) indicated the pixels affected by a decreasing trend (site degradation). (**e**) Indicates vegetation trends at two points of interest: blue negative trend, red stable trend. (**f**,**g**) Field survey highlighted that this decreasing trend is mainly linked to the parasite attack which in the last 5 years strongly affected the pinus tree and dramatically damaged the area.

The reliability of the analytical results obtained from the Fisher-Shannon approach was assessed by comparisons with field surveys and independent data analyses. In fact, the results obtained from the statistical analysis herein conducted fit well with the results obtained from the processing of Sentinel 2 data jointly carried out by CNR and Carabinieri [35] and shown in Figure 9; the dark grey pixels (in Figure 9d) indicate the areas affected by a decreasing trend (site degradation) as a resulting effect of the parasite attacking the pinus trees; the white pixels are related to areas involved in increasing trend, mainly linked to agricultural activities. Finally, Figure 9f,g, acquired during the field survey, clearly provide evidence of the macroscopic effect of the *Toumeyella parvicornis* on the pinus trees. An example of this behaviour, i.e., grey and white pixels, related to decreasing and increasing trends, is shown in Figure 9e, where the blue and red lines depicted the maximum NDVI over time as obtained from Sentinel 2 data for the pixels indicated by the blue and red triangles, respectively.

The inner dynamic of the vegetation of Appia Antica Park seems quite stable, and this was confirmed by in situ analysis. This site is mainly involved in and characterised by agricultural activities that were conducted systematically maintaining the same cultivation types for the whole period of our analysis [35]. The behaviour of the inner vegetation appears without anomalous dynamics, because the area was not involved in significant changes of vegetation status as well as of the land use and land cover as it can be seen from the Corine land cover updates (freely available online in the framework of the Copernicus initiative; see, for example, CORINE Land Cover—Copernicus Land Monitoring Service).

**Figure 10:**
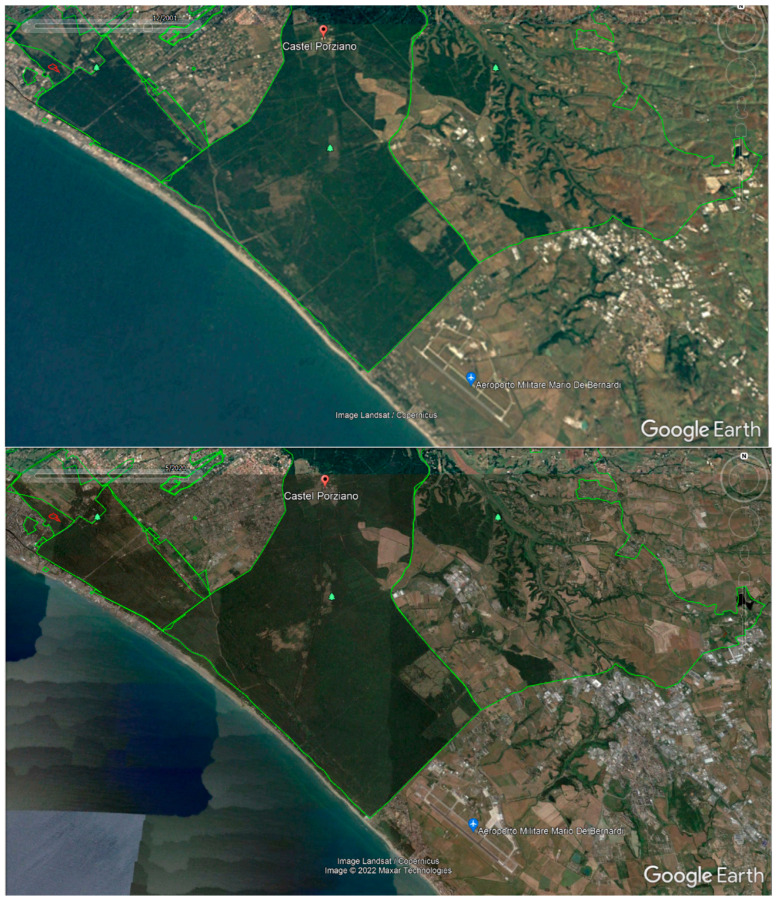
Castel Porziano from GE high-resolution images, which show that from 2000 to 2020 no significant changes occurred (Google Earth Pro courtesy).

Castel Porziano, instead, presents FIM and Shannon Entropy values in the middle between those of Castel Volturno and Appia, except for the evapotranspiration. Comparison with independent data sets [35] can confirm that from 2000 to 2020 the area was quite stable, as it can be seen from Figure 10, where the Google Earth satellite pictures at higher resolution do not show particular changes in land cover.

## 6. Conclusions

The vegetation of three study areas from the Central (Appia Ancient Park and Castel Porziano) to the Southern (Castel Volturno) parts of Italy were analyzed. The study areas were periurban and specifically selected as key in improving environmental quality: in fact, they are rich in biodiversity and allow urban areas to be more sustainable, helping to combat climate change and make cities more comfortable, as recently strongly emphasized by the COVID-19 pandemic emergency.

Thus, for each site we focused on the detrended series, since this represents the inner time variability of the vegetation not influenced by external meteoclimatic factors.

Results of our analyses highlighted that the (i) trend is characterized by an oscillatory behaviour that explains the seasonal cycles of the series, very likely linked with the meteo-climatic variability, (ii) detrended series, although apparently noisy, would represent the inner time dynamics of the series that might be not influenced by external driving mechanisms.

Among the sites investigated, Castel Volturno was characterized by the highest Shannon entropy power and the lowest FIM that indicate a low level of order and organization of the vegetation indices for this site. Independent analyses and field survey highlighted that Castel Volturno is strongly affected by a parasite, the *Toumeyella parvicornis*, which has been provoking dramatic damage to the Pinus trees in recent years.

Our results could contribute to the definition of methods suitable for an early diagnosis of deterioration trends, and create operational tools for multiscale, multisensor, multitemporal monitoring of biophysical parameters relating to the state of vegetation.

Our future work will be in the application of robust statistical analyses to satellite time series to define, for example, indicators devised to assess and monitor land degradation that can be applied at different spatial and temporal scales using different satellite time series (MODIS along with Sentinel 2 data sets). In fact, the use of EO-based indicators is particularly relevant because RS-derived data have been shown to be useful across many fields, and at the local to global levels using data freely available from NASA (MODIS, TM) and ESA (Sentinel 1–5).

## Figures and Tables

**Table 1:** Meteoclimatic and vegetational characteristics of the investigated sites.

Study Area	Castel Volturno	Castel Porziano	Appia Park
Longitude	14°1′45″ E	12°23′36″ E	12°31′55″ E
Latitude	40°56′1″ N	41°42′35″ N	41°49′40″ N
Area [km^2^]	19	85	96
Annual precipitation [mm]	1078	878	878
Annual mean temp. [°C]	15.5	15.8	15.8
Climate system by the Köppen-Geiger climate classification	Hot-summer Mediterranean climate, Csa	Csa	Csa
Vegetation description	268 hectares The site is mainly characterized by the presence of woods holm oak, pine forests with Pinus pinea, and a nucleus of retro-dunal hygrophilous vegetation.	2300 hectares The site is mainly characterized by the presence of holm oak (261 hectares), cork oak wood (460 hectares), and stone pine forest (750 hectares) The woods alternate with clearings and natural grasslands.	4580 hectares: It is a mosaic of different environments: large spaces intended for cultivation and extensive grazing are interrupted by uncultivated areas; residual wooded strips, where agricultural exploitation has not arrived or has long since ceased; ditches with the presence of riparian vegetation and some wet areas.

**Table 2:** Values of minimum MDL.

	Castel Volturno	Castel Porziano	Appia
ET	5	7	11
EVI	5	10	10
LAI	5	7	9
NDVI	5	9	10

## Supplementary Materials

The following supporting information can be downloaded at: https://www.mdpi.com/article/10.3390/e24121784/s1.
