# Supplementary material for: Exploring Long-Term Anomalies in the Vegetation Cover of Peri-Urban Parks Using the Fisher-Shannon Method
Source: Entropy (Basel). 2022 Dec 6;24(12):1784. doi: 10.3390/e24121784 (PMC9777874; doi:10.3390/e24121784)
Supplement: Supplementary file 1 [file entropy-24-01784-s001.zip › entropy-1966083-supplementary.pdf]

Description of the datasets

MOD16A2.006: Terra Net Evapotranspiration: It can be used to calculate regional water and energy balance, soil water status. With long-term ET data, the effects of changes in climate, land use, and ecosystems disturbances (e.g. wildfires and insect outbreaks) on regional water resources and land surface energy change can be quantified [Running et al., 2017]. MOD16 is a *VI* model based on the Penman-Monteith equation driven by MODIS data, and global meteorological reanalysis from the Modern-Era Retrospective analysis for Research and Applications (MERRA) (Mu et al., 2011). *ET* can be estimated summing up soil evaporation (E_s), canopy evaporation (E_c), and canopy transpiration (T_c).

$$ET = E_S + E_C + T_C \tag{1}$$

The radiation arriving to the soil can be partitioned between canopy and soil surface using the fraction of photosynthetically active radiation (f_{PAR}) assuming that f_{PAR} and canopy cover (f_c) are equal. MOD16 also considers the pixel wet surface fraction (f_w), calculated as a function of relative humidity (*RH*; when *RH* is higher than 70%, $f_w = RH^4$), representing the fraction of vegetation and soil covered by water (Laipelt et al., 2021). Estimations of E_s, E_c and T_c are given by Equations 2 to 4, respectively.

$$E_S = f_w \frac{\Delta A_S + \frac{(1-f_C)\rho_a C_P (e_S - e_a)}{r_a^S}}{\Delta + \gamma \frac{r_S^S}{r_a^S}} + RH^{\frac{(e_S - e_a)}{\beta sm}} (1 - f_w) \frac{\Delta A_S + \frac{(1-f_C)\rho_a C_P (e_S - e_a)}{r_a^S}}{\Delta + \gamma \frac{r_S^S}{r_a^S}} \tag{2}$$

$$E_c = f_w \frac{\Delta A_S + \frac{f_C \rho_a C_P (e_S - e_a)}{r_a^{wc}}}{\Delta + \gamma \frac{r_S^{wc}}{r_a^S}} \tag{3}$$

$$T_c = (1 - f_w) \frac{\Delta A_c + \frac{f_C \rho_a C_P (e_S - e_a)}{r_a^t}}{\Delta + \gamma (1 + \frac{r_S^t}{r_a^S})} \tag{4}$$

where Δ is the gradient of the saturation vapor pressure–temperature, A_s and A_c are the available energy to the soil and canopy, respectively, γ is the psychrometric constant, β_{sm} is a parameter related to the soil moisture constraint, r^s_s and r^s_a are the surface and aerodynamic resistance for the soil surface, r^{wc}_s and r^{wc}_a are the surface and aerodynamic resistance for the wet canopy evaporation and r^t_s and r^t_a are the surface and aerodynamic resistance for the canopy transpiration (Laipelt et al., 2021).

MOD13Q1.006 Terra Vegetation Indices: MODIS vegetation indices are derived from atmospherically-corrected reflectance in the red, near-infrared, and blue wavebands; the normalized difference vegetation index (NDVI), and the enhanced vegetation index (EVI), which minimizes canopy-soil variations and improves sensitivity over dense vegetation conditions [Didan, 2015]:

$$NDVI = \frac{(\rho_{NIR} - \rho_{red})}{(\rho_{NIR} + \rho_{red})}$$

where ρ_{NIR}, ρ_{red} are the surface reflectance over the near-infrared (NIR) and red bands of MODIS. The NDVI is well correlated with vegetation cover, vegetation canopy, vegetation dynamics, biomass, and leaf area index and often considered as the vegetation proxy [Huete et al., 1999; Kumari et al., 2020; and Huete et al., 1993].

Similar to Normalized Difference Vegetation Index (NDVI), Landsat Enhanced Vegetation Index (EVI) can also quantify vegetation greenness in addition to correcting for some atmospheric conditions and canopy background noise (e.g. highly variable aerosol conditions, such as smoke from biomass burning) which makes it more sensitive in areas with dense vegetation [Kumari et al., 2021; and Huete et al., 1999]:

$$EVI = G(\frac{\rho_{NIR} - \rho_{red}}{\rho_{NIR} + C_1(\rho_{red}) - C_1(\rho_{blue}) + L})$$

where G is a gain factor (G = 2.5), C_1 = 6 and C_2 = 7.5 are the aerosol coefficients. L adjustment factor to correct the effects induced by canopy background, and ρ values represents the surface reflectance which are atmospherically corrected.

MOD15A2H.006: Terra Leaf Area Index/FPAR: LAI is defined as the one-sided green leaf area per unit ground area and is used for calculating surface photosynthesis, evapotranspiration, and net primary production. It is calculated based on the fraction of photosynthetically active radiation absorbed by green vegetation [Myneni et al., 2015].

MOD11A2.006 Terra Land Surface Temperature and Emissivity: The Land Surface Temperature (LST) daily data are retrieved by the day/night algorithm where, daytime and nighttime LSTs and surface emissivities are retrieved from pairs of day and night MODIS observations in seven TIR bands [Wan et al., 2015].

TerraClimate: Monthly Climate and Climatic Water Balance for Global Terrestrial Surfaces, University of Idaho: It incorporates reference evapotranspiration, precipitation, temperature, and interpolated plant extractable soil water capacity. It uses climatically aided interpolation, combining high-spatial resolution climatological normals from the WorldClim dataset, with coarser spatial resolution, but time-varying data from CRU Ts4.0 and the Japanese 55-year Reanalysis (JRA55) [Abatzoglou et al., 2018].

Table S1. General description of bands used in this study from the datasets available in the cloud storage of Google Earth Engine.

**Collection Snippet**	**Dataset Availability**	**Bands**						**RESOLUTION**	
		**Name**	**Units**	**in**	**ax**	**cale**	**Description**	**Tempor**	**Spatial [m]**

								al [day]	
ee.ImageCollection("MODIS/006/MOD16A2")	2001-2022	ET	kg/m^2/8day	32767	2700	.1	Total evapotranspiration	8	500
ee.ImageCollection("MODIS/006/MOD13Q1")	2000-2022	NDVI	[-]	2000	0000	.0001	Normalized Difference Vegetation Index	16	250
		EVI	[-]	2000	0000	.0001	Enhanced Vegetation Index	16	250
ee.ImageCollection("MODIS/006/MOD15A2H")	2000-2022	Lai_500m	sq. meter/sq. meter		00	.1	Leaf Area Index	8	500
ee.ImageCollection("MODIS/006/MOD11A2")	2000-2022	LST_Day_1km	Kelvin	500	5535	.02	Day land surface temperature	8	1000
ee.ImageCollection("IDAHO_EPSCOR/TERRACLIMATE")	1958-2020	pdsi	[-]	4317*	418*	.01	Palmer Drought Severity Index	monthly	4638.3
